# Dynamic m⁶A methylation during bovine preadipocyte differentiation and functional implication of the m⁶A writer METTL14

**DOI:** 10.1186/s12864-026-12858-w

**Published:** 2026-04-20

**Authors:** Dianqi Zhang, Jiawei Du, Ni Gao, Hehan Qiu, Huaxuan Li, Chugang Mei, Linsen Zan

**Affiliations:** 1 College of Animal Science and Technology, Northwest A&F University, Yangling, Shaanxi 712100 P. R. China; 2 National Beef Cattle Improvement Center, Northwest A&F University, Yangling, Shaanxi 712100 P. R. China; 3 College of Grassland Agriculture, Northwest A&F University, Yangling, Shaanxi 712100 PR China

**Keywords:** Bovine, *METTL14*, MeRIP-seq, Adipogenesis

## Abstract

As a fundamental mechanism of post-transcriptional epigenetic regulation, RNA modifications have attracted increasing research interest in recent years. N6-methyladenosine (m^6^A), the most abundant internal RNA modification, plays a crucial role in regulating multiple stages of mRNA metabolism including processing, transport, translation, and degradation. The differentiation of bovine preadipocytes into mature adipocytes is regulated at multiple levels including transcriptional, post-transcription, translation, and post-translation levels. However, the precise regulatory mechanism of m^6^A modification during bovine adipogenesis remains poorly understood. In this study, we conducted MeRIP-seq and RNA-seq analyses across three stages of bovine preadipocyte differentiation—pre-differentiation (day 0, D0), early differentiation (day 2, D2), and late differentiation (day 8, D8)—to characterize the dynamic landscape of m⁶A methylation during adipogenesis. The analysis identified 400 genes exhibiting significant changes in both m⁶A modification levels and mRNA expression during the differentiation phase (D0–D2), while 592 genes exhibited such changes during the lipid accumulation phase (D2–D8). These results indicate that m⁶A modification likely contributes to adipogenesis by modulating the expression of these genes. To further investigate the functional relevance of the m⁶A machinery, we knocked down the m⁶A writer METTL14, a core component of the methyltransferase complex, and performed RNA-seq analysis. Integrative analysis of the differentially expressed genes (DEGs) with the stage-specific expression profile revealed a set of candidate genes potentially regulated by METTL14 that participate in early preadipocyte differentiation and late-stage lipid accumulation. These findings provide new evidence for the molecular mechanisms underlying m⁶A modification during bovine adipogenesis and highlight a functional contribution of the m⁶A writer METTL14.

**Supplementary Information:**

The online version contains supplementary material available at 10.1186/s12864-026-12858-w.

## Introduction

Beef is valued by consumers for its high-quality protein content and essential fatty acids. Intramuscular fat (IMF) content critically influences key beef quality attributes, including flavor, tenderness, and juiciness [1, 2]. High-IMF beef is generally considered a premium product and frequently experiences market shortages. The deposition of IMF is a complex biological process regulated at multiple levels, including transcriptional, post-transcriptional, and translational control [3]. Current molecular breeding research in beef cattle mainly concentrates on identifying and functionally characterizing key genes, whereas the role of RNA modifications on trait improvement remains largely unexplored.

Structurally, RNA exhibits diverse chemical modifications, with over 170 distinct types identified to date [4]. Among these, m^6^A is the most prevalent internal modification in mRNA and has emerged as one of the most extensively studied RNA epigenetic marks. It plays a critical role in cellular development and functional maintenance by regulating processes such as pre-mRNA alternative splicing, nuclear export, translation efficiency, stability, and degradation [5–8]. The discovery that FTO (Fat mass and obesity-associated protein) can demethylate m^6^A revealed that, similar to DNA methylation, m⁶A is dynamically reversible, establishing it as a major focus in RNA epigenetics. The deposition of m^6^A is catalyzed by a methyltransferase complex (MTC) with METTL3 (Methyltransferase 3) and METTL14 (Methyltransferase 14) serving as its core enzymatic subunits [9, 10]. In addition to its core subunits, the methyltransferase complex comprises auxiliary components such as WTAP (Wilms Tumor 1-Associating Protein), RBM15/15B (RNA Binding Motif Protein 15/15B), ZC3H13 (Zinc Finger CCCH-Type Containing 13), and VIRMA (Vir-Like m^6^A Methyltransferase Associated), which collectively regulate m^6^A deposition and its specific localization [11–15].

The currently known m^6^A demethylases, which belong to the Fe(II)/α-ketoglutarate-dependent dioxygenase superfamily of the AlkB family, include FTO and ALKBH5 (AlkB Homolog 5) [16–19]. These two demethylases exhibit distinct functional mechanisms due to their structural differences [20]. Proteins that directly recognize m^6^A and mediate its functional consequences are referred to as “readers.” The YTH domain family, comprising YTHDF1 (YTH N6-Methyladenosine RNA Binding Protein F1), YTHDF2 (YTH N6-Methyladenosine RNA Binding Protein F2), YTHDF3 (YTH N6-Methyladenosine RNA Binding Protein F3), YTHDC1 (YTH N6-Methyladenosine RNA Binding Protein C1), and YTHDC2 (YTH N6-Methyladenosine RNA Binding Protein C2), represents the most thoroughly characterized group of m^6^A readers. YTHDC1 is predominantly nuclear and regulates RNA splicing, whereas the remaining members exhibit high cytoplasmic expression and are involved in mRNA stability, translation, and degradation [21, 22]. Beyond the YTH family, several other m⁶A reader proteins have also been identified and characterized. For example, the IGF2BP family (IGF2BP1/2/3, Insulin-like Growth Factor 2 mRNA Binding Protein 1/2/3) recognizes m⁶A modifications to enhance the stability and translation efficiency of their target mRNAs, consequently affecting cellular processes such as metabolism and tumorigenesis. Members of the HNRNP family, including HNRNPA2B1 (Heterogeneous Nuclear Ribonucleoprotein A2/B1), HNRNPC (Heterogeneous Nuclear Ribonucleoprotein C), and HNRNPG (Heterogeneous Nuclear Ribonucleoprotein G), primarily regulate pre-mRNA splicing and alternative transcription. Additionally, the eIF3 complex promotes cap-independent translation initiation through its interaction with m⁶A modified sites [23].

Research has demonstrated that m⁶A modification is extensively involved in diverse physiological and pathological processes, such as glucose metabolism [24–26], type 2 diabetes [27–29], tumorigenesis and progression [30, 31], muscle cell differentiation, and cardiac function regulation [32, 33]. Recent studies have established that m⁶A modification plays an important regulatory role in adipogenesis. MeRIP-seq analyses have revealed how m⁶A-modifying proteins influence adipocyte differentiation by modulating m⁶A levels on key adipogenic genes [34]. Genome-wide association studies (GWAS) indicate that variations in the FTO gene are significantly correlated with obesity [35, 36]. FTO was later characterized as an RNA demethylase responsible for catalyzing the removal of m⁶A. The knockdown of FTO significantly increased m⁶A levels in mRNA [16]. During adipogenesis, PPARG (Peroxisome Proliferator-Activated Receptor Gamma) acts as a master regulator of adipocyte differentiation [37]. FTO promotes adipogenesis by enhancing *PPARG* expression through the reduction of m⁶A modification on *PPARG* transcripts [38]. METTL3 has also been implicated in adipogenesis, as its knockdown enhances *JAK1* (Janus Kinase 1) mRNA stability by reducing m⁶A modification, which subsequently upregulates *C/EBPβ* (CCAAT Enhancer Binding Protein Beta) expression and promotes adipocyte differentiation [39]. Beyond mammalian adipogenesis, m⁶A modification is increasingly recognized for its association with economically important traits in livestock. Dynamic m⁶A changes have been observed during follicular and testicular development in pigs [40]. Moreover, MeRIP-seq analyses in chicken muscle suggest that m⁶A modification is associated with the regulation of fat deposition [41, 42]. In cattle, m⁶A regulates muscle cell differentiation by modulating MEF2C [43]. However, transcriptome-wide mapping of m⁶A during bovine preadipocyte differentiation remains limited, leaving an important research gap unaddressed.

This study characterizes m⁶A modification patterns during bovine preadipocyte differentiation and investigates the potential regulatory relevance of m⁶A dynamics. Furthermore, we functionally examine the contribution of the m⁶A writer METTL14 and identify candidate genes potentially regulated by METTL14 to advance our understanding of m⁶A-associated regulation in bovine adipogenesis.

## Materials and methods

### Ethics statement

All animal experiments were conducted in strict accordance with institutional, national, and international guidelines for the care and use of animals in research. In particular, all procedures adhered to Directive 2010/63/EU of the European Parliament on the protection of animals used for scientific purposes. The experimental cattle were obtained from the breeding farm of Northwest A&F University. All procedures involving animals were reviewed and approved by the Institutional Animal Care and Use Committee (IACUC) of Northwest A&F University (Ethics Committee Name: Institutional Animal Care and Use Committee of Northwest A&F University; Approval Number: DK2021042). Prior to tissue collection, the animals were gently restrained and rendered unconscious by electrical stunning. Loss of consciousness was confirmed by the presence of tonic spasm, fixed eyeballs, absence of the corneal reflex, and lack of struggling. The animals were then immediately exsanguinated by severing the carotid arteries and jugular veins, and tissues were collected promptly thereafter. No pharmacological anaesthetic was used in this study in order to avoid potential interference of anaesthetic residues with primary cell isolation, cell viability, and subsequent experimental results.

### Isolation and culture of bovine preadipocytes

Primary bovine preadipocytes were isolated from the perirenal adipose tissue of three 1-day-old Qinchuan calves raised under identical feeding and management conditions, following a previously established method from our laboratory [44]. Briefly, adipose tissue was collected under sterile conditions, washed three times with PBS containing antibiotics, and carefully cleared of connective tissue and blood vessels. The tissue was minced into approximately 1 mm³ fragments and digested with type I collagenase at 37 °C for 90 min with gentle agitation. The digestion was terminated by adding an equal volume of complete culture medium, and the cell suspension was filtered through 200 μm and 70 μm cell strainers. After centrifugation (1000 rpm, 10 min), erythrocytes were removed using red blood cell lysis buffer. The cells were washed twice with serum-free medium, resuspended in basal medium, and seeded into T75 culture flasks (NEST Biotechnology, China). Cells were maintained at 37 °C in a humidified incubator with 5% CO₂, and the medium was replaced after 24 h to remove non-adherent cells. Upon reaching approximately 90% confluence, the medium was replaced with Induction Medium I. After two days, the medium was changed to Induction Medium II to promote adipocyte differentiation. The basal medium contained 90% DMEM/F12, 10% fetal bovine serum (NEST Biotechnology, China), and 1% penicillin-streptomycin solution. Induction Medium I was prepared by supplementing the basal medium with 0.5 mM 3-isobutyl-1-methylxanthine (IBMX, Beijing Solarbio, China), 5 µg/mL insulin, 1 µM dexamethasone, and 1 µM rosiglitazone. Induction Medium II was formulated by adding only 5 µg/mL insulin to the basal medium. Cells were passaged using 0.25% trypsin (NEST Biotechnology, China) digestion.

### Cell identification

Preadipocytes at 90% confluence were subjected to Pref-1 immunofluorescence staining (Supplementary Fig. 1A) using the protocol previously established in our laboratory [45].

### Quantification of m⁶A content by LC-MS/MS

Total RNA was isolated from bovine preadipocytes at various time points during differentiation. mRNA was purified with the BeyoMag™ Magnetic Beads mRNA Purification Kit (Beyotime Biotechnology, China). A 20 µL digestion reaction containing 100 ng of purified mRNA, 2 U nuclease P1, 25 mM NaCl, and 1.5 mM ZnCl₂ was incubated at 37 °C for 2 h. The reaction mixture was then supplemented with 3 µL of 1 M NH₄HCO₃ and 1 U alkaline phosphatase (New England Biolabs, China), followed by a further 2-hour incubation at 37 °C. After the reaction, the mixture was diluted five-fold with deionized water and centrifuged at 10,000 rpm and 4 °C for 5 min. The supernatant was collected for further analysis. The resulting samples were analyzed on an Agilent 1290 Infinity II–6470 LC–MS/MS system to quantify m⁶A and adenosine (A) levels and calculate the m⁶A/A ratio.

### Quantification of m⁶A RNA methylation (colorimetric method)

The m⁶A content was measured with the EpiQuik™ m⁶A RNA Methylation Quantification Kit (Colorimetric) from Epigentek (USA). Total RNA was extracted from bovine preadipocytes collected at different days of differentiation, and 300 ng of this RNA was used for each m⁶A assay. The RNA was first immobilized on the well surfaces with a high-efficiency binding solution to ensure stable attachment while preserving its native conformation. m⁶A sites were then specifically captured by a bound antibody. A detection antibody was introduced, forming an immunocomplex with the capture antibody. After adding a signal enhancement solution to amplify the detection signal, a color development solution was applied. This immunocomplex catalyzed a colorimetric reaction where the intensity of the resulting blue color was directly proportional to the m⁶A content. The reaction was stopped with a stop solution once the positive control wells developed a moderate blue color, changing the solution from blue to yellow. The absorbance at 450 nm (OD value) was finally recorded using a microplate reader. The absolute m⁶A content or relative percentage in the samples was determined from the linear relationship between the OD values and a standard curve, allowing for the quantification of RNA m⁶A modification levels.

### Dot blot

The purified mRNA was diluted to 100 ng/µL, denatured at 95 °C for 3 min, and immediately chilled on ice. A 1 µL aliquot of the sample was applied to a Hybond-N⁺ nylon membrane (GE Healthcare, USA) and air-dried at room temperature followed by UV cross-linking for 20 min. After that, the membrane was blocked with rapid blocking buffer (Beyotime Biotechnology, China) for 30 min at room temperature, and incubated overnight at 4 °C with an m⁶A-specific primary antibody (Synaptic Systems GmbH, Germany). The next day, the membrane was incubated with an HRP-conjugated secondary antibody for 2 h at room temperature. After incubation, a chemiluminescent substrate (UElandy, Suzhou, China) was applied for signal detection, and signals were detected and imaged using a protein imaging system (BIO-RAD, USA). After that, the membrane was stained for 30 min with 0.02% methylene blue solution prepared in 0.3 M sodium acetate, rinsed with deionized water, and imaged under white light to confirm uniform RNA loading.

### RNA isolation and sequencing library construction

Total RNA was isolated from bovine preadipocytes at differentiation D0, D2 and D8 using the TRIzol reagent (Accurate Biotechnology, China). The RNA samples were subsequently sent to Novogene Co., Ltd. (China) for both MeRIP-seq and RNA-seq analyses. RNA integrity and concentration were assessed using an Agilent 2100 Bioanalyzer (Agilent) and a SimpliNano spectrophotometer (GE Healthcare). Fragmented mRNA (~ 100 nt) was immunoprecipitated with an m^6^A antibody (Synaptic Systems) at 4 °C for 2 h. Library construction was then performed using the NEBNext^®^ Ultra™ II RNA Library Prep Kit for Illumina^®^ (New England Biolabs). The resulting libraries were sequenced on an Illumina platform using a paired-end 150 bp (PE150) strategy. Three biological replicates were included for each experimental group.

### RNA-seq data analysis

Raw sequencing reads were subjected to quality control using fastp (v0.19.11), which removed adapter sequences, low-quality reads, and reads with excessive N bases to yield high-quality clean reads. The Q20, Q30, and GC content of these clean reads were then calculated to assess data quality. All downstream analyses were performed using these high-quality reads. The reference genome (ARS-UCD1.2) was indexed using HISAT2 (v2.0.5), and the clean reads were aligned to it. Read counts for each gene were quantified using Subread (v1.5.0-p3). The FPKM (Fragments Per Kilobase of transcript per Million mapped reads) for each gene was calculated from its length and the corresponding read counts. Differential expression analysis between experimental groups was performed with the DESeq2 R package (v1.14.1). The DEGs were defined by the thresholds |log₂FC| ≥ 1 and an adjusted *P*-value (padj) < 0.05.

### MeRIP-Seq data analysis

We identified transcriptome-wide m⁶A-enriched regions (peaks) using the R package exomePeak (v2.16.0), applying a significance threshold of FDR < 0.05. Input RNA-seq data were used as the background control to improve the accuracy of peak detection. The detected peaks were annotated according to genomic features, and genes containing peaks located in exonic regions were designated as m⁶A peak-associated genes. To characterize the distribution of m⁶A modifications across transcripts, each peak was assigned to one of five mRNA regions: transcription start site (TSS), 5′UTR, coding sequence (CDS), stop codon region (STOP), or 3′UTR. We then visualized the distribution and regional enrichment of these peaks using the R package Guitar, which highlights positional trends of m⁶A along transcripts. Using HOMER software (v4.9.1), we performed motif analysis on sequences from significantly enriched m⁶A peaks to identify consensus motifs. Differential m⁶A peak analysis between comparison groups was performed using exomePeak (parameters: *P* ≤ 0.05 and |fold change| ≥ 1.5), and the identified differential peaks were subsequently annotated.

### cDNA synthesis and qRT-PCR

The cDNA library was constructed with reagent Evo M-MLVRT Mix Kit with gDNA Clean for qPCR Ver.2 (Accurate Biotechnology, China). Quantitative real-time polymerase chain reaction (qRT-PCR) was then performed using PerfectStart^®^ Green qPCR SuperMix (TransGen Biotech, China). Relative gene expression levels were calculated using the 2^-ΔΔCt method. ACTB was used as the internal reference gene. Primer sequences are listed in Supplementary Table 1.

### m^6^A immunoprecipitation quantitative real-time PCR (m^6^A-IP-qPCR)

Total RNA was isolated from bovine preadipocytes on differentiation D0, D2, and D8. Primers targeting approximately 300 bp regions flanking the m^6^A modification sites predicted by SRAMP were designed [46, 47], with sequences listed in Supplementary Table 1. RNA immunoprecipitation was performed using the CUT&RUN m^6^A RNA Enrichment (MeRIP) Kit (Epigentek, New York, USA). Magnetic beads conjugated with RNA were incubated with an anti-m^6^A antibody, followed by digestion of unbound RNA fragments using an RNA cleavage enzyme. The immunoprecipitated RNA was eluted from the beads and reverse transcribed. The qRT-PCR was then conducted using PerfectStart^®^ Green qPCR SuperMix. Relative quantification was performed using the 2^-ΔΔCt method, with the input RNA sample as the control.

### H&E staining

*Longissimus dorsi* muscle samples were collected from Qinchuan beef Cattle (QC) and Wagyu. Following paraformaldehyde fixation, the specimens were embedded in paraffin, sectioned, and stained with hematoxylin and eosin. Images were captured using an Olympus microscope (Olympus Corporation, Tokyo, Japan).

### Oil Red O Staining

Adipocytes differentiated for 8 days were washed twice with PBS and fixed in paraformaldehyde. After 30 min, the cells were stained with Oil Red O staining kit (Beijing Solarbio Science & Technology Co., Ltd., China). Excess dye was removed by washing, and images were captured using an Olympus microscope (Olympus Corporation, Tokyo, Japan).

### BODIPY staining

Adipocytes differentiated for 8 days were fixed and then incubated with a 10µM BODIPY staining solution. Following a PBS wash, the cells were stained with DAPI for 10 min. After removing excess dye with further washes, images were acquired randomly using an EVOS™ Auto 2 Imaging System (Thermo Fisher Scientific, USA).

### Triglyceride (TG) quantification

Adipocytes subjected to differentiation for D0, D2 and D8 were collected, and their TG content was quantified according to established methods [48].

### Statistical analysis and data visualization

All data are reported as the mean ± SD from three independent biological replicates. Statistical analyses and graph generation were performed with GraphPad Prism 8.0.2 (GraphPad Software, USA), and data were visualized using RStudio (version 4.3.2). Differences among three or more groups were assessed by one-way analysis of variance (ANOVA), whereas comparisons between two groups were performed using an independent samples t-test. Statistical significance was defined as *P* < 0.05, and a threshold of *P* < 0.01 indicated high statistical significance.

## Result

### m⁶A modification is associated with IMF deposition and adipocyte differentiation

**Fig. 1:**
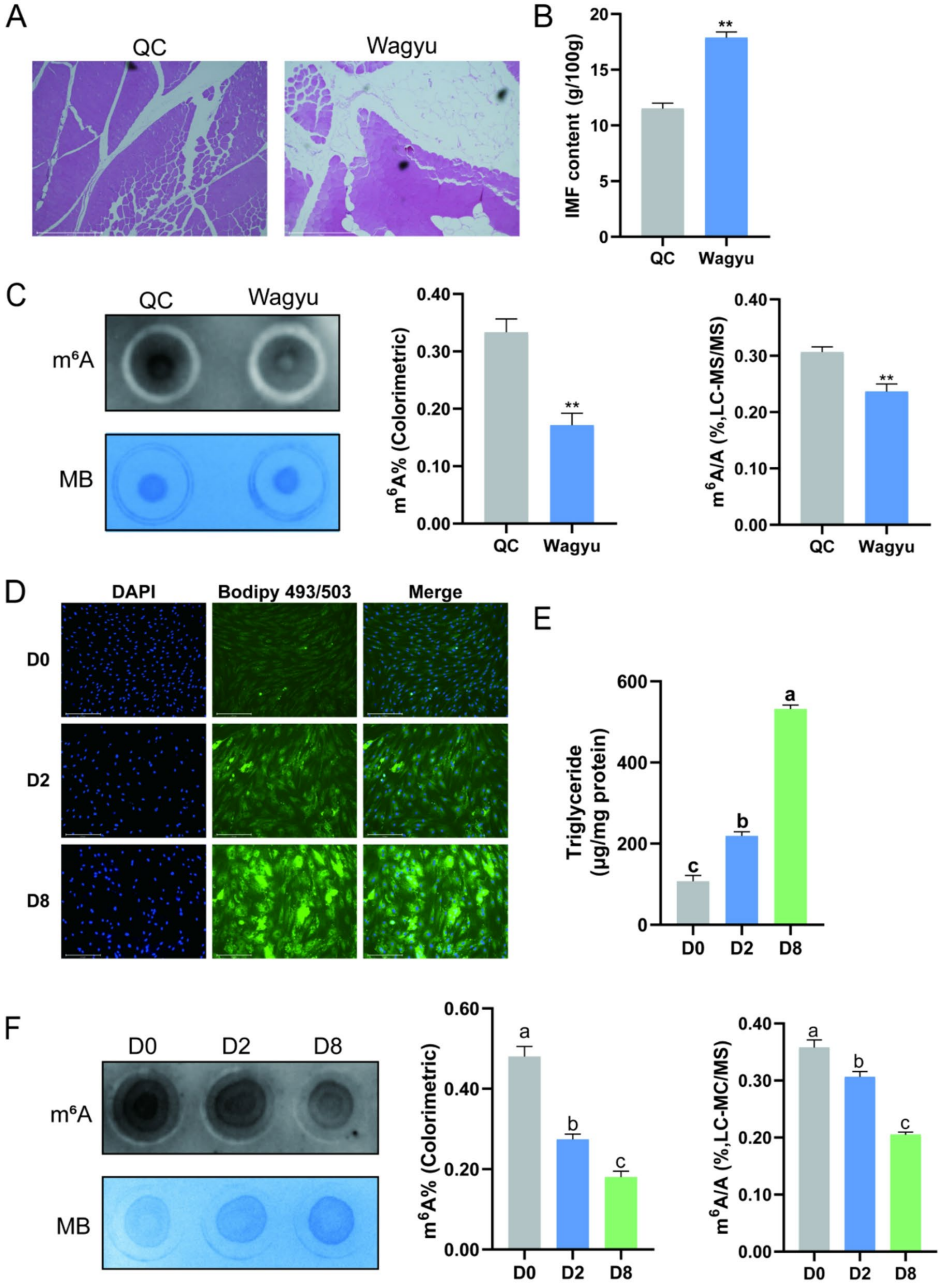
m⁶A modification is associated with IMF deposition and preadipocyte differentiation. **A** H&E staining of QC and Wagyu (10×, scale bar: 500 μm). **B** IMF content in the bovine *Longissimus dorsi* muscle. **C** (Left) Dot blot analysis of QC and Wagyu; (Middle and Right) m^6^A levels detected by colorimetric assay and LC-MS/MS, respectively. **D** Results of Bodipy staining (200×, scale bar: 275 μm). **E** Statistical data for cellular TG. **F** (Left) Dot blot analysis of bovine preadipocytes at D0, D2 and D8 of differentiation; (Middle and Right) m^6^A levels detected by colorimetric assay and LC-MS/MS, respectively. Data are presented as mean ± SD (*n* = 3). Statistical significance between two groups was determined using an independent-samples t-test, while comparisons among three or more groups were analyzed by one-way ANOVA. ** indicates *P* < 0.01. Different lowercase letters indicate *P* < 0.05

To investigate the potential role of m^6^A modification in adipogenesis, we first quantified the IMF content in QC and Wagyu cattle. Wagyu exhibited a significantly higher IMF content than QC (Fig. 1A-B). However, dot blot analysis revealed that the overall m^6^A modification level in QC *Longissimus dorsi* muscle tissue was significantly higher than in Wagyu. This observation was further supported by subsequent LC–MS/MS and colorimetric assays which consistently showed higher m⁶A levels in QC samples. (Fig. 1C). Furthermore, lipid droplet accumulation and TG content were examined at three time points during the ex vivo differentiation of preadipocytes: pre-differentiation (D0), early differentiation (D2), and late differentiation (D8). Both lipid droplet accumulation and TG content increased significantly during adipocyte differentiation. (Fig. 1D-E). Simultaneously, m^6^A levels decreased significantly during the differentiation process (Fig. 1F). These findings suggest that m⁶A modification is associated with adipogenesis and may contribute to the differences in IMF deposition observed between QC and Wagyu.

### The m⁶A writer METTL14 positively promotes adipogenesis

**Fig. 2:**
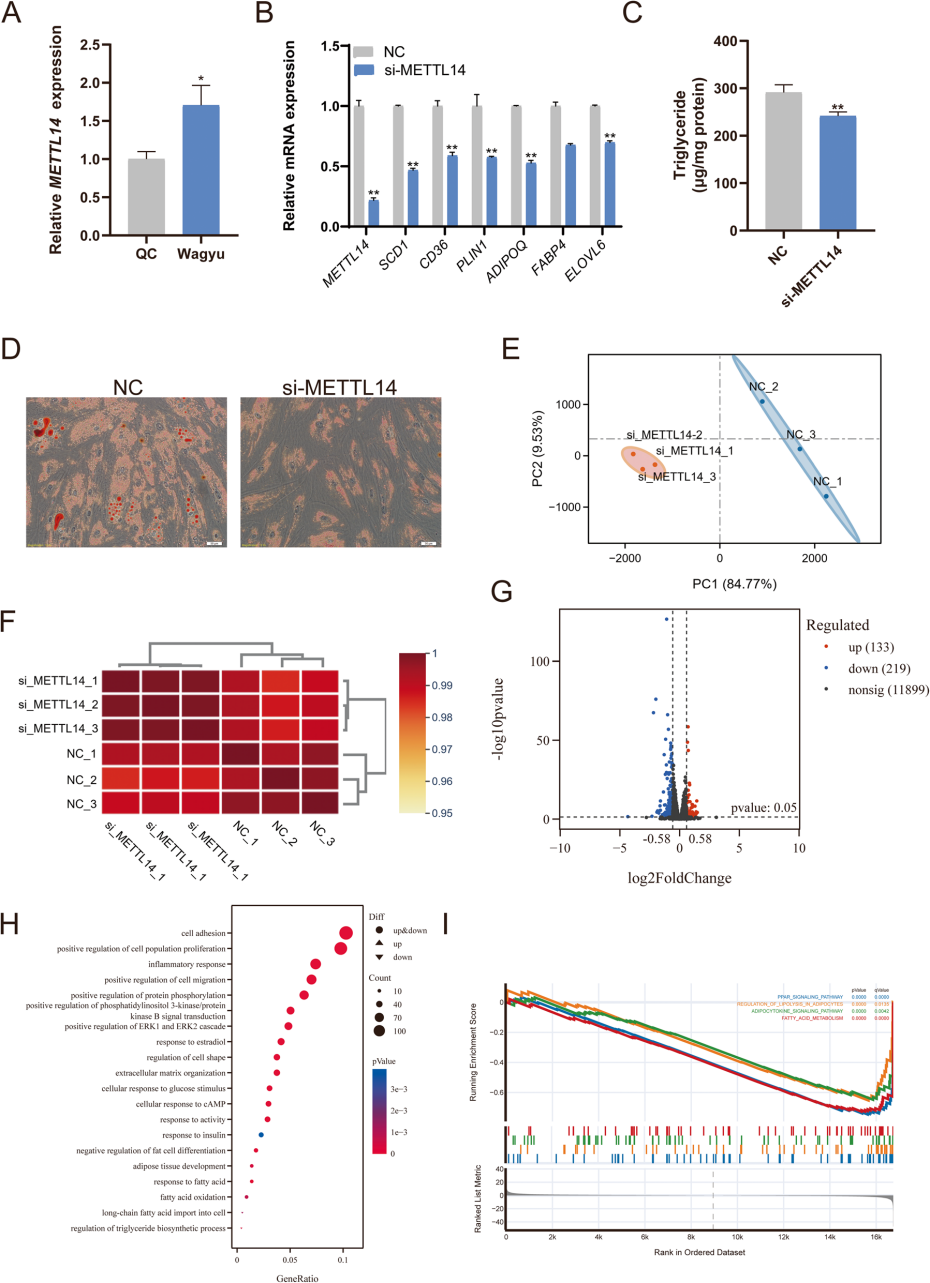
*METTL14* positively promotes adipogenesis. **A** Relative mRNA expression levels of *METTL14* in the *Longissimus dorsi* muscle of QC and Wagyu. **B** Relative mRNA expression levels following METTL14 knockdown in bovine adipocytes. **C** Quantification of intracellular TG content after METTL14 knockdown. **D** Oil Red O staining showing lipid accumulation after METTL14 knockdown (magnification ×200; scale bar = 50 μm). **E** PCA plot of RNA-seq samples. **F** Heatmap of Pearson correlation coefficients among RNA-seq samples. **G** Volcano plot of DEGs following METTL14 knockdown. **H** GO enrichment analysis of DEGs. **I** GSEA of pathways affected by METTL14 knockdown. Data are presented as mean ± SD (*n* = 3). Statistical significance between two groups was determined using an independent-samples t-test. * *P* < 0.05, ** *P* < 0.01

METTL14, a core component of the m^6^A methyltransferase complex, plays an important role in regulating lipid deposition and adipogenesis. Comparison of *METTL14* expression in *Longissimus dorsi* muscle tissue revealed significantly lower levels in QC than in Wagyu. (Fig. 2A). We designed and transfected siRNA targeting *METTL14* into bovine preadipocytes, resulting in an approximately 80% reduction in *METTL14* expression eight days post-transfection. This knockdown markedly inhibited lipid droplet accumulation and significantly reduced the expression of adipogenesis-related genes (Fig. 2B-D). To further investigate the regulatory role of METTL14 in adipogenesis, we performed RNA-seq on the *METTL14*-knockdown adipocytes. The biological replicates showed high Pearson correlation coefficients, and principal component analysis (PCA) confirmed clear and consistent clustering (Fig. 2E-F). Differential expression analysis identified 352 DEGs, with 133 upregulated and 219 downregulated (Fig. 2G). Gene Ontology (GO) enrichment analysis revealed that the DEGs were significantly enriched in multiple adipogenesis-related biological processes, including negative regulation of fat cell differentiation, fatty acid oxidation, and regulation of triglyceride biosynthesis (Fig. 2H). Gene Set Enrichment Analysis (GSEA) further revealed that *METTL14* knockdown markedly suppressed key signaling pathways involved in lipid metabolism, such as the PPAR signaling pathway and fatty acid metabolism (Fig. 2I).

### Overview of m^6^A modification levels during bovine preadipocyte differentiation

**Fig. 3:**
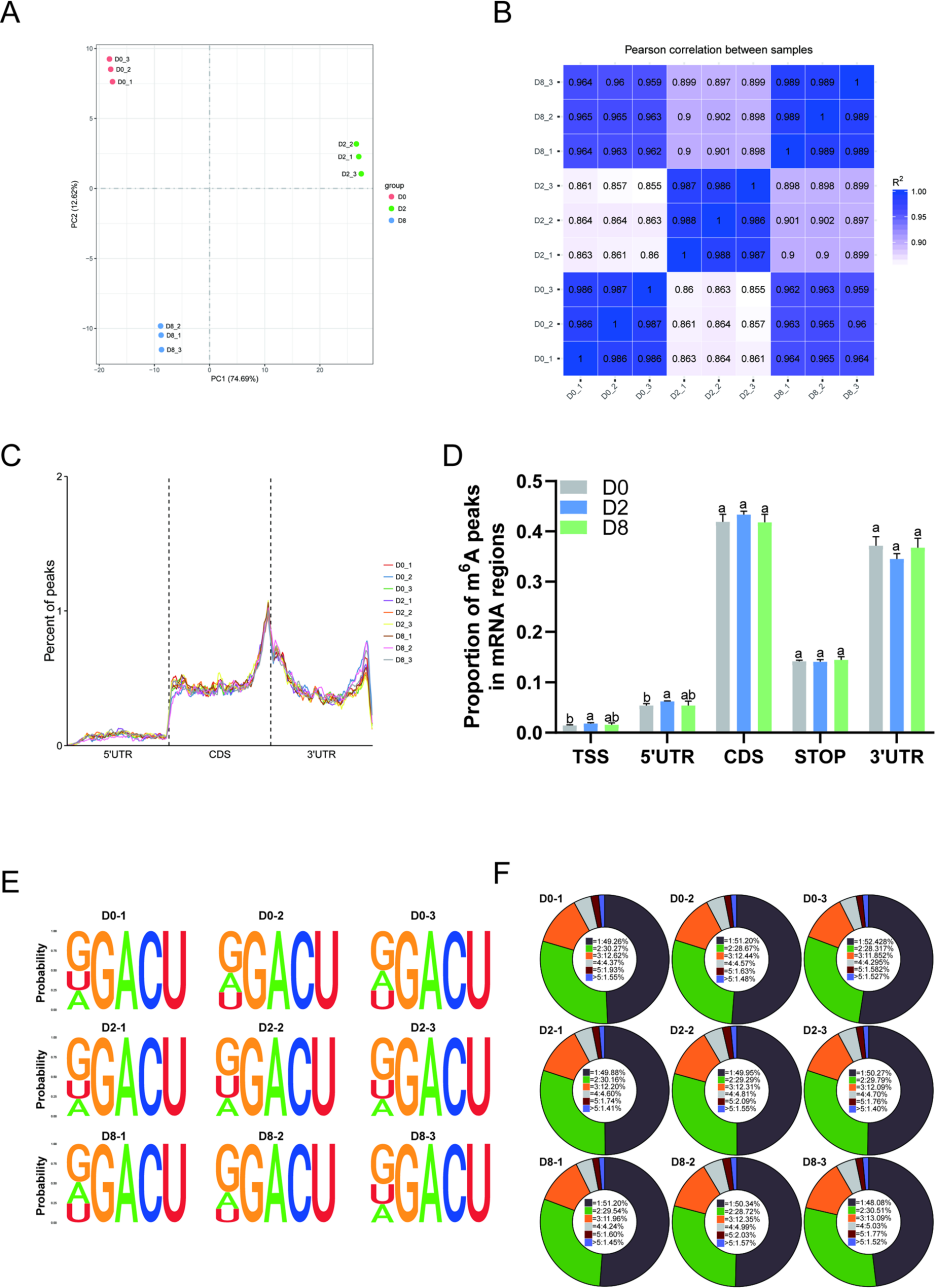
Overview of m^6^A modification levels during bovine preadipocyte differentiation. **A** PCA plot of MeRIP-seq sample. **B** Heatmap showing Pearson correlation coefficients among biological replicates. **C** Transcript-wide distribution pattern of m⁶A peaks across mRNA regions. **D** Proportion of m^6^A peaks distributed across mRNA regions, different lowercase letters indicate significant differences, with *P* < 0.05. **E** Sequence logo representation of enriched m⁶A consensus motifs identified from MeRIP-seq peaks at D0, D2, and D8. **F** Percentage distribution of transcripts containing different numbers of m⁶A peaks

To investigate m^6^A modification patterns during adipogenesis, RNA was extracted from adipocytes at D0, D2, and D8 for MeRIP-seq analysis. PCA was initially performed to assess the overall distribution of the samples. The three biological replicates from each time point clustered together, demonstrating the stability of the sequencing data (Fig. 3A). Pearson correlation analysis further confirmed strong correlations among samples within the same group, supporting their suitability for subsequent experimental analyses (Fig. 3B). Over 95% of the clean reads from sequencing were successfully aligned to the bovine reference genome, with uniquely mapped reads also exceeding 95%, confirming the high quality and reliability of high-throughput sequencing data (Table 1). To characterize the transcriptome-wide distribution of m⁶A modifications, we analyzed the chromosomal localization of m⁶A peaks. These peaks were broadly distributed across all chromosomes without obvious chromosomal bias (Supplementary Fig. 1B). Mapping of m⁶A peaks to mRNA structural regions indicated that m⁶A modifications were predominantly enriched in CDS and 3’UTRs, with a notable concentration near the stop codon. Conversely, only minimal enrichment was observed in 5’UTRs and around the start codon (Fig. 3C-D). The enriched m⁶A peaks contained the canonical DRACH (D = A/G/U, R = A/G, H = A/C/U) consensus motif (Fig. 3E). During adipogenic differentiation of bovine preadipocytes, each transcript contained an average of approximately 0.57–0.64 m⁶A peaks. Among the m⁶A-modified transcripts, each exhibited approximately 1.83–1.92 peaks on average (Table 2). Further quantification revealed that over 75% of m⁶A-modified mRNAs contained 1–2 m⁶A peaks, whereas nearly 20% possessed at least 3 m⁶A peaks (Fig. 3F).

**Table 1:** Summary of MeRIP-seq data and read-alignment statistics in bovine preadipocytes

Sample	Clean reads	Mapped reads	Unique Mapped reads
D0_1_Input	48,636,748	46,769,864(97.03%)	46,758,484(97.01%)
D0_2_Input	43,910,582	41,881,692(97.14%)	41,870,274(97.11%)
D0_3_Input	44,414,600	42,805,912(97.18%)	42,795,040(97.15%)
D2_1_Input	46,089,750	44,310,424(97.06%)	44,299,066(97.03%)
D2_2_Input	54,185,810	52,131,713(97.03%)	52,119,305(97.01%)
D2_3_Input	48,230,390	46,345,045(97.06%)	46,333,879(97.03%)
D8_1_Input	47,466,226	45,397,178(96.89%)	45,384,413(96.86%)
D8_2_Input	53,042,180	50,586,200(97.14%)	50,571,604(97.11%)
D8_3_Input	59,248,858	56,593,108(97.14%)	56,578,205(97.11%)
D0_1_IP	47,298,158	44,935,052(95.78%)	44,917,036(95.75%)
D0_2_IP	41,958,522	39,842,828(95.82%)	39,825,035(95.78%)
D0_3_IP	43,287,094	41,163,491(95.77%)	41,144,993(95.72%)
D2_1_IP	45,263,494	42,928,017(95.62%)	42,909,328(95.58%)
D2_2_IP	40,856,514	38,826,142(95.77%)	38,808,554(95.73%)
D2_3_IP	39,592,792	37,611,511(95.76%)	37,596,384(95.72%)
D8_1_IP	41,012,922	39,000,311(95.77%)	38,982,276(95.73%)
D8_2_IP	43,860,594	41,758,693(96.09%)	41,739,555(96.05%)
D8_3_IP	41,315,590	39,219,562(95.72%)	39,203,837(95.68%)

**Table 2:** Number of m^6^A peaks during bovine preadipocyte differentiation

Samples	Total transcripts	Total m^6^A transcripts	Total m^6^A peaks	m^6^A peaks per m^6^A transcripts	m^6^A peaks per transcripts
D0_1_IP	27,607	8881	16,833	1.90	0.61
D0_2_IP	27,607	8785	16,381	1.86	0.59
D0_3_IP	27,607	8528	15,679	1.83	0.57
D2_1_IP	27,607	8788	16,489	1.87	0.60
D2_2_IP	27,607	8895	16,918	1.90	0.61
D2_3_IP	27,607	8741	16,366	1.87	0.59
D8_1_IP	27,607	8711	16,076	1.85	0.58
D8_2_IP	27,607	8849	16,831	1.90	0.61
D8_3_IP	27,607	9197	17,666	1.92	0.64

### Analysis of DEGs across distinct stages of bovine preadipocyte differentiation

**Fig. 4:**
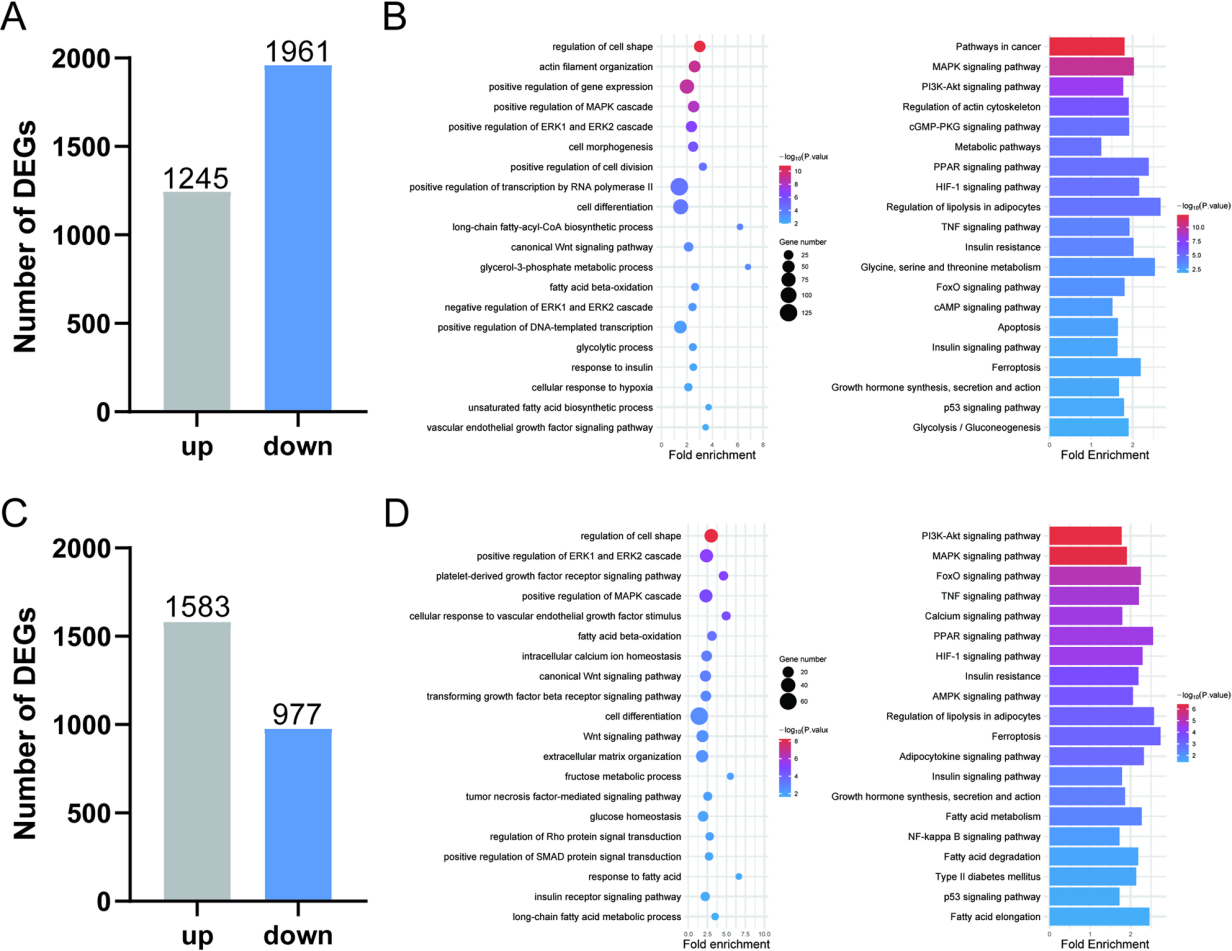
Analysis of DEGs across distinct stages of bovine preadipocyte differentiation. **A** Number of DEGs at D2 of adipocyte differentiation compared to D0. **B** GO and KEGG enrichment analysis of DEGs (D2 vs. D0). **C** Number of DEGs at D8 of adipocyte differentiation compared to D2. **D** GO and KEGG enrichment analysis of DEGs (D8 vs. D2)

During adipocyte differentiation, significant changes occur at the transcriptional level. Initially, DEGs in the early differentiation stage of adipocytes were analyzed, identifying a total of 1,245 significantly upregulated genes and 1,961 significantly downregulated genes (adjusted *P* < 0.05, |log₂FC| ≥ 1) (Fig. 4A). GO enrichment analysis revealed that these DEGs were primarily involved in biological processes such as regulation of cell shape, long-chain fatty-acyl-CoA biosynthetic process, and response to insulin. Furthermore, KEGG pathway enrichment analysis showed that the DEGs were significantly enriched in key signaling pathways, including the MAPK signaling pathway, PI3K-Akt signaling pathway, and PPAR signaling pathway (Fig. 4B). These results indicate the successful establishment of an adipocyte differentiation model. During the early differentiation phase, DEGs promote the initiation of adipocyte differentiation by regulating processes such as cell morphology, fatty acid synthesis, and insulin response, thereby activating multiple signaling pathways closely associated with adipogenesis.

During the lipid deposition stage, the gene expression profile of adipocytes continued to undergo dynamic changes, with 1,583 genes being significantly upregulated and 977 genes significantly downregulated (adjusted *P* < 0.05, |log₂FC| ≥ 1) (Fig. 4C). GO enrichment analysis demonstrated that the DEGs at this stage were mainly involved in metabolism-related processes, including fatty acid beta-oxidation, glucose homeostasis, and response to fatty acid. KEGG analysis indicated significant enrichment in signaling pathways such as the PI3K-Akt signaling pathway, PPAR signaling pathway, and Adipocytokine signaling pathway (Fig. 4D). These findings indicate that during the lipid accumulation stage, adipocytes predominantly regulate the molecular regulatory network responsible for lipid droplet formation and maturation through modulation of pathways related to fatty acid metabolism and energy homeostasis.

Collectively, the DEGs across distinct differentiation stages exhibited stage-specific functional characteristics: the early phase was primarily associated with the initiation of differentiation signals and cell fate determination, whereas the later phase focused on the precise regulation of lipid synthesis and energy metabolism.

### Analysis of DMGs at different stages of bovine preadipocyte differentiation

**Fig. 5:**
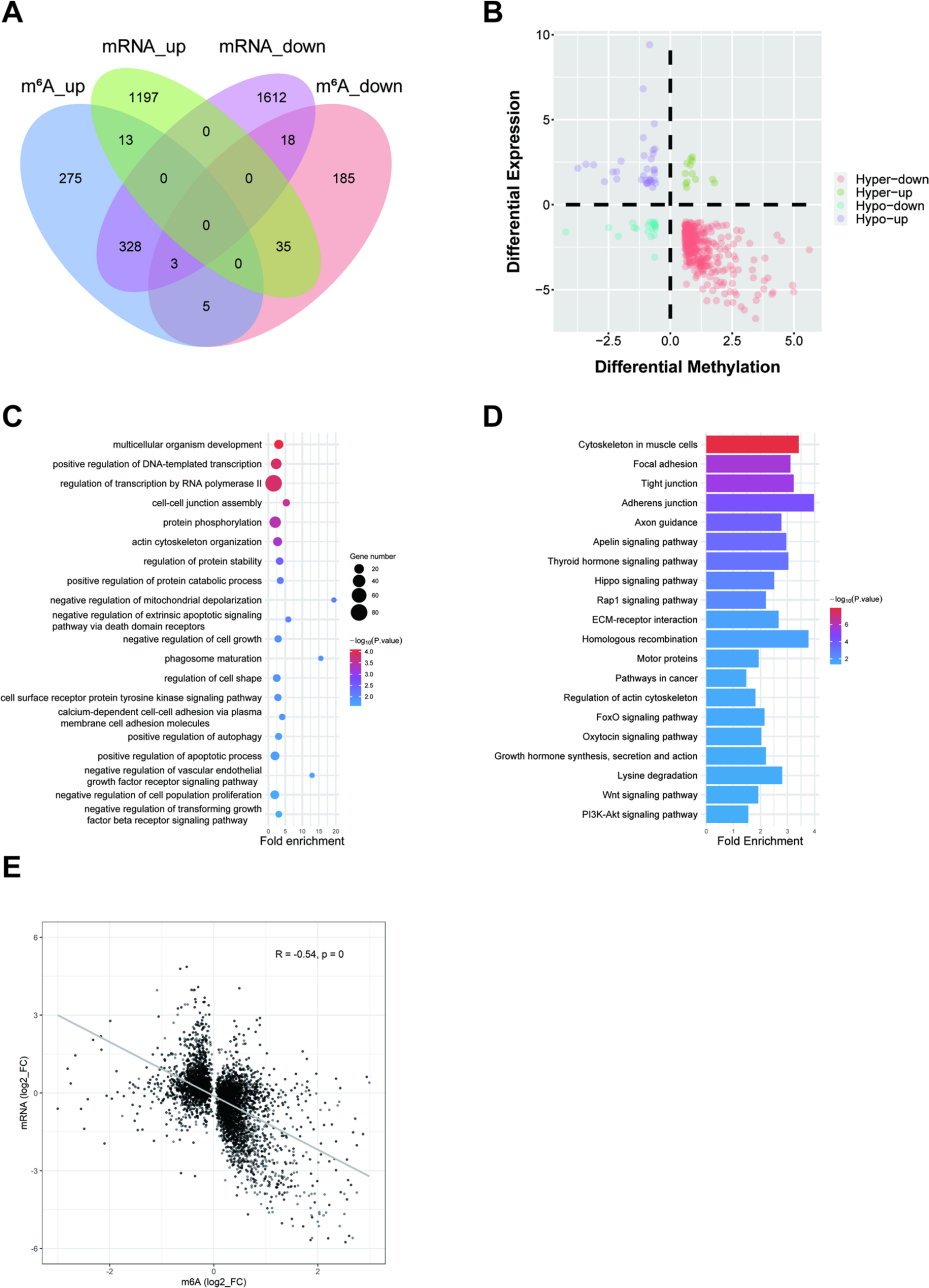
Analysis of DMGs at different stages of bovine preadipocyte differentiation. **A** Overlapping genes between DMGs and DEGs at day D2 versus day D0. **B** The number of genes displaying both significant differential m^6^A modification and significant differential mRNA expression levels at day D2 relative to day D0. **C** GO enrichment analysis of DMGs (D2 vs. D0). **D** KEGG enrichment analysis of DMGs (D2 vs. D0). **E** Pearson correlation analysis between the fold change in m⁶A methylation and the fold change in mRNA expression (D2 vs. D0)

To investigate the dynamic changes and functional roles of m^6^A modification during adipocyte differentiation, we first compared m^6^A modification profiles between the early differentiation stage (D2) and the undifferentiated state (D0). This analysis identified 4,767 differential m^6^A peaks, including 1,129 statistically significant peaks (FC > 1.5, *P* < 0.05) corresponding to 862 differentially methylated genes (DMGs). Among these DMGs, 624 genes exhibited significantly increased m^6^A modification levels, while 246 showed significantly decreased levels. Notably, 8 genes simultaneously contained both upregulated and downregulated modification sites (Fig. 5A), suggesting the presence of multiple m^6^A sites with independent regulatory functions. GO enrichment analysis revealed that these DMGs were primarily involved in biological processes including multicellular organism development, negative regulation of cell growth, and regulation of cell shape (Fig. 5C). KEGG pathway analysis indicated significant enrichment in the Hippo signaling pathway, FoxO signaling pathway, and PI3K-Akt signaling pathway (Fig. 5D). These findings suggest that m^6^A modification plays an important role during early preadipocyte differentiation by regulating pathways related to cell morphology, proliferation, and development.

**Fig. 6:**
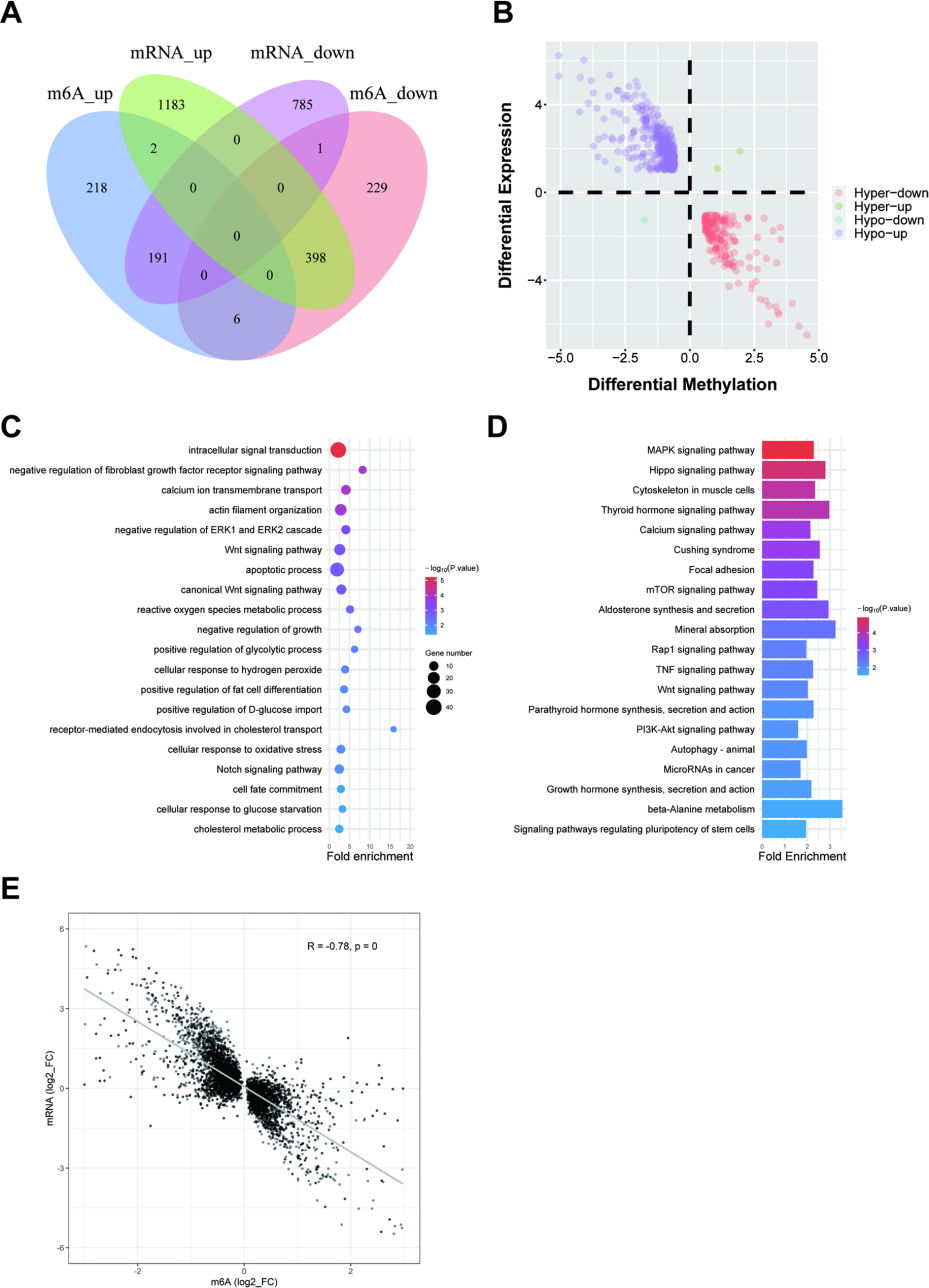
Analysis of DMGs at different stages of bovine preadipocyte differentiation. **A** Overlapping genes between DMGs and DEGs at day D8 versus day D2. **B** The number of genes displaying both significant differential m^6^A modification and significant differential mRNA expression levels at day D8 relative to day D2. **C** GO enrichment analysis of DMGs (D8 vs. D2). **D** KEGG enrichment analysis of DMGs (D8 vs. D2). **E** Pearson correlation analysis between the fold change in m⁶A methylation and the fold change in mRNA expression (D8 vs. D2)

During the lipid deposition stage (D8 vs. D2), we identified 6,774 differential m^6^A peaks, including 1,956 statistically significant peaks (FC > 1.5, *P* < 0.05) corresponding to 1,045 DMGs. Among these, 417 genes showed significantly increased methylation levels while 634 exhibited decreased levels, with 6 genes containing both upregulated and downregulated modification sites simultaneously (Fig. 6A). GO enrichment analysis showed that these DMGs were mainly involved in intracellular signal transduction, positive regulation of glycolytic process, and positive regulation of fat cell differentiation (Fig. 6C). KEGG analysis revealed significant enrichment in pathways including the MAPK signaling pathway, Cushing syndrome, and autophagy in animals (Fig. 6D). These results revealed that m^6^A modification contributes to adipogenesis and the maintenance of energy homeostasis during lipid accumulation by regulating pathways involved in signal transduction, glucose metabolism, and autophagy.

To explore the relationship between m^6^A modification and gene expression, we integrated the data on m^6^A modification levels and mRNA expression changes. Between D2 and D0, 400 genes showed significant changes at both m^6^A and mRNA levels, suggesting their potential involvement in early preadipocyte differentiation via m^6^A modification. Quadrant analysis identified 13 Hyper_up (hypermethylation and upregulated expression), 331 Hyper_down (hypermethylated and downregulated expression), 35 Hypo_up (hypomethylated and upregulated expression), and 21 Hypo_down genes (hypomethylated and downregulated expression) (Fig. 5B). Between D8 and D2, we identified 592 genes with simultaneous significant changes, comprising 2 Hyper_up, 191 Hyper_down, 398 Hypo_up, and 1 Hypo_down genes (Fig. 6B). These results suggest that these genes may contribute to regulating bovine preadipocyte differentiation through m^6^A modification.

Finally, Pearson correlation analysis was performed to evaluate the relationship between m^6^A modification levels and mRNA expression changes. The results indicated a moderate positive correlation (|r| > 0.5) between D2 and D0 (Fig. 5E). This correlation strengthened further between D8 and D2 (|r| > 0.7) (Fig. 6E), suggesting that the association of m^6^A modification on gene expression becomes more pronounced during differentiation.

**Fig. 7:**
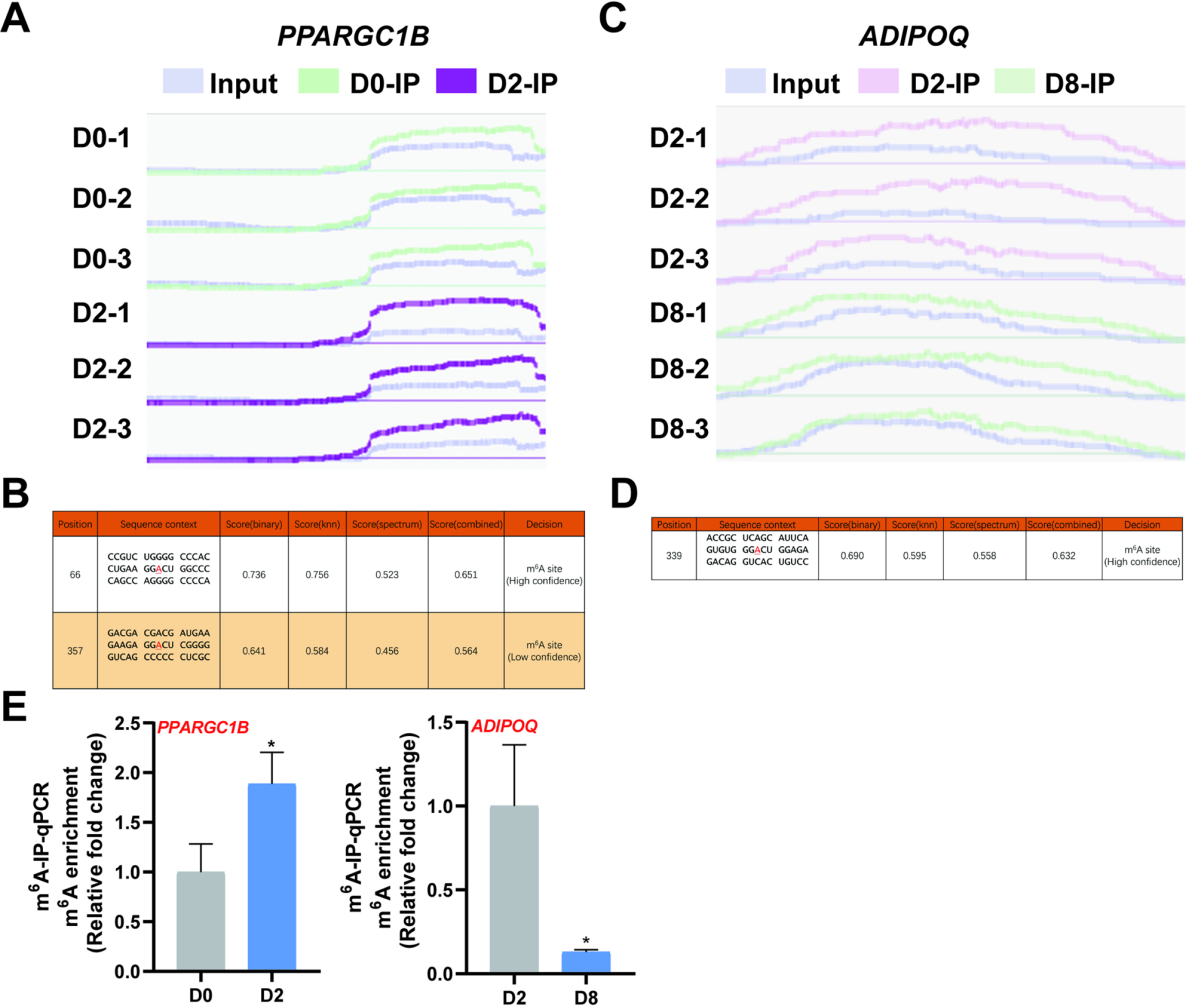
Prediction and Validation of m⁶A Methylation Sites for Selected Genes. **A** IGV visualization shows the m⁶A methylation site distribution in the *PPARGC1B* gene. **B** An online database predicts potential m⁶A sites in *PPARGC1B*. **C** The distribution of m⁶A methylation sites in the *ADIPOQ* gene is visualized using IGV. **D** Potential m⁶A sites in *ADIPOQ* were predicted with an online database. **E** m⁶A-IP-qPCR validated the enrichment of m⁶A modifications in *PPARGC1B* and *ADIPOQ*

To identify key functional target genes, we intersected DEGs from *METTL14* interference experiments with candidate gene sets from the quadrant analyses of both differentiation stages. In differentiating preadipocytes, 19 candidate genes were screened (Supplementary Table 2), including *PPARGC1B* (PPARG Coactivator 1 Beta) and *NKD1* (NKD Inhibitor Of Wnt Signaling Pathway 1), which are closely associated with preadipocyte differentiation. At the adipogenesis stage, 43 genes were identified (Supplementary Table 3), including *PLPP3* (Phospholipid Phosphatase 3) and *ADIPOQ* (Adiponectin), which directly participate in lipid deposition. The m^6^A methylation peaks of these genes were then visualized in the IGV browser (Fig. 7A-B), and their methylation sites were predicted using an online platform (http://www.cuilab.cn/sramp) (Fig. 7C-D). The m^6^A-IP-qPCR assays validated the m^6^A methylation levels of the relevant genes (Fig. 7E). These genes not only reflect the dynamic regulatory characteristics of m^6^A modification but also play crucial roles in adipogenesis, potentially representing candidate functional targets associated with the METTL14–m^6^A regulatory axis.

## Discussion

Qinchuan cattle rank among China’s five principal indigenous yellow cattle breeds, valued for their meat quality and robust adaptability [49]. However, key economic traits—especially IMF content—remain lower than those of elite foreign breeds such as Wagyu [50]. Our findings confirm that IMF content in QC is significantly lower than in Wagyu. Therefore, this study aims to explore potential epigenetic mechanisms associated with IMF deposition in QC, with the goal of providing insights for meat quality improvement.

As the first identified internal mRNA modification [51], m^6^A plays a critical role in regulating mRNA stability and function [52]. Recent evidence indicates that m^6^A modification has been implicated in the regulation of various economic traits in domestic animals [53, 54]. Comparison of m⁶A modification levels in IMF from QC and Wagyu revealed significantly lower global m⁶A levels in the high-fat-content Wagyu compared with QC. Interestingly, although METTL14 expression was significantly higher in Wagyu compared with QC, the global m⁶A level was lower in Wagyu. At first glance, this observation appears inconsistent with the established role of METTL14 as a core component of the m⁶A methyltransferase complex. However, global m⁶A abundance represents the integrated outcome of multiple regulatory factors, including methyltransferases, demethylases, transcriptome composition, and transcript-specific methylation dynamics. This apparent discrepancy likely reflects differences between complex tissue composition and the in vitro adipocyte differentiation system. Intramuscular tissue represents a heterogeneous environment dominated by myofibers, whereas adipocytes account for only a small fraction of total RNA. Therefore, global m⁶A levels measured in muscle largely reflect composite transcriptomic contributions from multiple cell types rather than adipocytes alone. In contrast, the in vitro differentiation model captures intrinsic m⁶A dynamics within a relatively homogeneous adipocyte population. These findings suggest that global m⁶A abundance may not serve as a direct linear indicator of fat deposition capacity, and that adipogenesis may be more strongly influenced by transcript-specific m⁶A remodeling rather than overall methylation quantity. Therefore, changes in the expression of a single writer component do not necessarily lead to proportional alterations in total m⁶A levels. Moreover, biological effects related to IMF deposition are more likely mediated by methylation changes at specific functional transcripts rather than by overall m⁶A abundance. Studies in pigs and chickens have also reported divergent or species-specific trends. A negative correlation was observed between m^6^A modification levels and fat deposition capacity in the intramuscular adipose tissue of Jinhua pigs (fat-type breed) and Landrace pigs (lean-type breed) [55]. Conversely, chicken leg muscle, which possesses higher IMF content, exhibited increased m^6^A modification levels [41], a phenomenon potentially linked to the generally low IMF in chickens [56]. Furthermore, we measured TG content and m^6^A modification at three key time points during adipocyte differentiation. The m^6^A levels decreased significantly as lipid droplets progressively accumulated during differentiation. However, studies in 3T3-L1 cells reported the opposite trend [57]. This discrepancy may arise from species-specific differences in adipocyte differentiation regulatory networks and the expression patterns of m⁶A-related enzymes, suggesting that m⁶A exerts a complex, context-dependent role in fat deposition. This context dependency may arise from multiple factors. First, the expression levels and relative activities of m⁶A writers, erasers, and readers vary across species and tissues, potentially leading to distinct regulatory outcomes. Second, species-specific differences in m⁶A motif distribution and sequence architecture may influence m⁶A deposition efficiency and reader recognition. For example, previous studies reported GGACA as the predominant motif in chicken intramuscular fat, whereas GGACU was enriched in porcine muscle. In our bovine data, we observed broader enrichment of (G/U/A)GACU motifs. Although these motifs all conform to the canonical DRACH consensus, subtle variations in sequence context may modulate methyltransferase recruitment or reader-binding affinity, thereby contributing to species-specific regulatory effects.

*METTL14*, a core component of the m⁶A methyltransferase complex, forms a heterodimer with *METTL3* to co-catalyze mRNA adenosine methylation at the N6 position [58]. It primarily recognizes and binds RNA substrates, thereby enhancing the specificity and stability of the methylation process. METTL14 contributes to the regulation of global m⁶A levels and influences gene expression post-transcriptionally by modulating mRNA splicing, stability, nuclear export, and translation efficiency [59, 60]. Previous studies suggested that METTL14 participates in the regulation of preadipocyte fate through m⁶A-dependent mechanisms during adipogenesis, identifying it as an important epigenetic regulator of adipogenesis. The METTL3–METTL14–WTAP complex promotes preadipocyte proliferation and early differentiation by regulating mitotic clonal expansion in 3T3-L1 cells, thereby establishing a foundation for lipid droplet formation [61]. Reduced *METTL14* expression, in turn, inhibits adipogenesis by affecting genes such as *JAK2* (Janus Kinase 2) and *STAT3* (Signal Transducer And Activator of Transcription 3) [62]. Further in vivo studies demonstrated that adipose tissue-specific *METTL14* knockout significantly inhibits high-fat diet-induced obesity and adipose tissue expansion, whereas *METTL14* overexpression curtails lipolysis-related pathways via m⁶A modification and promotes fat deposition [63]. Our results similarly show that *METTL14* knockdown significantly inhibits adipogenesis and decreases the expression of key adipogenic genes. RNA-seq analysis, however, revealed that *METTL14* interference affected only a limited number of DEGs. To gain further insight, we therefore performed GSEA, which indicated that METTL14 interference markedly suppresses adipogenesis and activity of associated signaling pathways. These findings suggest that METTL14 may regulate adipocyte differentiation not primarily through large-scale transcriptional reprogramming, but via m⁶A-mediated post-transcriptional control of specific gene sets. The decrease in m⁶A modification caused by *METTL14* deficiency likely disrupts the stability and translation efficiency of adipogenesis-related mRNAs, ultimately impairing adipocyte differentiation and lipid droplet formation.

The development of MeRIP-seq has provided a powerful tool for mapping RNA m^6^A modifications and their functions [64]. However, the role of m^6^A in bovine adipogenesis remains largely unexplored, and the m^6^A modification landscape in these cells has not yet been characterized. To investigate the potential functions of m^6^A during bovine adipogenesis, we conducted transcriptome-wide m^6^A sequencing of bovine preadipocytes at differentiation D0, D2 and D8. Biological analysis indicated that DEGs were significantly enriched in biological processes associated with early preadipocyte differentiation and late lipid synthesis, supporting the biological relevance of our sequencing data. We found that 30.89–33.31% of transcripts contained m^6^A methylation during bovine preadipocyte differentiation. A comparable proportion (~ 30.77%) of m^6^A-modified transcripts was reported in human adipocytes using MeRIP-seq data [65]. Similarly, approximately 24.3–33.9% of transcripts were m^6^A-methylated in bovine muscle cells [15], aligning with our observations. Furthermore, over 75% of m^6^A-modified transcripts contained at least 1–2 peaks. Analysis of m^6^A peak distribution on mRNAs revealed enrichment within the coding sequence (CDS) and 3′ untranslated region (3′UTR). These modifications primarily occurred in the (G/U/A)GACU motif, which matches the mammalian DRACH consensus and is consistent with established distribution patterns [34, 66, 67]. The functional consequences of m^6^A modification are known to depend on its location. Modifications within the 3′UTR and CDS that are recognized by YTHDF1 enhance translation by increasing ribosome occupancy [68, 69], while those bound by YTHDF2 promote mRNA degradation via recruitment of the CCR4–NOT deadenylase complex [70]. YTHDF3 is proposed to play a dual role, cooperating with YTHDF1 to facilitate translation and with YTHDF2 to accelerate decay, thereby acting as a bridge in determining mRNA fate [21, 71]. IGF2BPs primarily bind to the CDS and 3′UTR; when m^6^A modifications are recognized by IGF2BP1/2/3, they recruit RNA-stabilizing complexes that protect mRNAs from degradation [72, 73].

Integrated analysis of MeRIP-seq and RNA-seq data revealed a significant moderate correlation between changes in gene m⁶A levels and gene expression during preadipocyte differentiation. A strong correlation between m⁶A level changes and gene expression changes emerged during adipogenesis, consistent with observations in goose embryonic muscle [74]. Interestingly, during bovine muscle differentiation, changes in mRNA expression levels showed no significant correlation with alterations in m⁶A modification [15]. During porcine embryonic muscle development, WGCNA identified two modules in which mRNA expression changes showed significant positive and negative correlations, respectively, with m⁶A level changes. These findings collectively indicate that the relationship between m⁶A modification and gene expression is highly species- and tissue-specific [75, 76].

To identify candidate differentially m⁶A-methylated genes potentially regulated by METTL14, we intersected genes from the differential expression analysis following *METTL14* knockdown with those derived from four-quadrant plots analysis of early and late differentiation stages. During the early differentiation stage, this approach highlighted genes including *SPP1* (Secreted Phosphoprotein 1), *DKK2* (Dickkopf Wnt Signaling Pathway Inhibitor 2), *WNT4* (Wnt Family Member 4), and *PPARGC1B* (PPARG Coactivator 1 Beta). Notably, previous studies in porcine mesenchymal stem cell adipogenic differentiation models have shown that *SPP1* overexpression inhibits the expression of adipogenic marker genes [77]. While *WNT4* represents a key WNT signaling pathway component, *DKK2* functions as its inhibitor [78], suggesting the involvement of the WNT pathway in early preadipocyte differentiation. WNT pathway activation is known to strongly suppress preadipocyte differentiation [79]. *PPARGC1B* has also been identified as associated with fat deposition [80]. During adipogenesis, we identified candidate genes associated with adipogenesis and lipid droplet accumulation, including *LPIN1* (Lipin 1), *LIPE* (Lipase E), *ADIPOQ*, and *PLIN4* (Perilipin 4). *LPIN1* plays an important role in TG synthesis and adipose tissue development, with its deficiency resulting in impaired adipose development [81]. Although LPIN1 functions as a phosphatidic acid phosphatase involved in triglyceride synthesis, it also contributes to adipocyte differentiation and lipid droplet homeostasis, where alterations in its activity or expression affect lipid accumulation in preadipocytes [82]. *ADIPOQ*, an adipocyte-secreted factor, serves not only as a mature adipocyte marker but also enhances adipogenic gene expression and increases lipid accumulation through autocrine/paracrine mechanisms [83]. Interestingly, one study found *ADIPOQ* promotes adipogenesis in an m⁶A-dependent manner during porcine intramuscular adipogenesis [84]. *PLIN4* is a member of the perilipin family of lipid droplet-associated proteins. Experimental evidence indicates that it is upregulated during mid-to-late stages of adipocyte differentiation, where it plays a key role in lipid droplet formation and the maintenance of cellular lipid homeostasis [85].

Finally, m⁶A modification levels of *PPARGC1B* and *ADIPOQ* were validated using m⁶A-IP-qPCR. Further investigation is required to determine how these genes regulate adipocyte differentiation and fat deposition through m⁶A modification. Based on our findings and observations across species, we propose that m⁶A modification may play distinct regulatory roles in fat deposition across different species.

## Conclusion

This study characterizes the m⁶A modification landscape across key stages of bovine preadipocyte differentiation. The m⁶A writer *METTL14* was found to play an important role in adipocyte lipid deposition. Furthermore, we identified candidate genes potentially influenced by METTL14-mediated m⁶A modification, such as *PPARGC1B* and *ADIPOQ*, which showed significant changes in both m⁶A levels and mRNA expression during differentiation. These findings provide new insights into the epigenetic regulation of adipogenesis in beef cattle, further supporting the involvement of m⁶A modification in lipid deposition. Furthermore, as aberrant lipid accumulation is a hallmark of obesity, our results suggest that m⁶A-mediated regulation of adipogenesis, particularly via METTL14, may offer insights into the epigenetic regulation of adipogenesis in broader metabolic contexts.

## Supplementary Information

Supplementary Material 1.

Supplementary Material 2.

Supplementary Material 3.

## Data Availability

The raw sequencing data have been deposited in the NCBI Sequence Read Archive (SRA) under the BioProject accession number PRJNA1364174. Additional data are available from the corresponding author upon reasonable request.

## Abbreviations

bp: Base Pair
CDS: Coding Sequence
cDNA: Complementary DNA
DEGs: Differentially Expressed Genes
DMGs: Differentially Methylated Genes
FC: Fold Change
FPKM: Fragments Per Kilobase of Transcript per Million Mapped Reads
GO: Gene Ontology
KEGG: Kyoto Encyclopedia of Genes and Genomes
GSEA: Gene Set Enrichment Analysis
GWAS: Genome-Wide Association Studies
H&E: Hematoxylin and Eosin
HRP: Horseradish Peroxidase
IBMX: 3-isobutyl-1-methylxanthine
IMF: Intramuscular fat
IP: Immunoprecipitation
LC-MS/MS: Liquid chromatography–tandem mass spectrometry
m⁶A: N6-methyladenosine
MeRIP-seq: methylated RNA immunoprecipitation sequencing
nt: Nucleotide
OD: Optical density
PCA: Principal component analysis
QC: Qinchuan beef cattle
rpm: Revolutions per minute
TSS: Transcription start site
UTR: Untranslated region
