# Supplementary material for: Dynamic m⁶A methylation during bovine preadipocyte differentiation and functional implication of the m⁶A writer METTL14
Source: BMC Genomics. 2026 Apr 20;27:511. doi: 10.1186/s12864-026-12858-w (PMC13224485; doi:10.1186/s12864-026-12858-w)
Supplement: Supplementary file 1 — Supplementary Material 1. [file 12864_2026_12858_MOESM1_ESM.docx]

| **Gene name** | **primer sequences** |
| --- | --- |
| q-METTL14 | F: TGTGTTTACGCAAGTGGGGT |
|  | R: CCCGTCTGTGCTACGCTTTA |
| q-SCD1 | F: TCCGACCTAAGAGCCGAGAA |
|  | R: AGCACAACAACAGGACACCA |
| q-CD36 | F: CAGCACTTGAGGCAGACACA |
|  | R: AAAAAGTGCAAGGCCACCAG |
| q-PLIN1 | F: ACCTGGTTGGTTTCTGAGAAGT |
|  | R: CGCCCTTCTCGTAGGCATT |
| q-ADIPOQ | F: TGGAGAAGCCGCTTATGTGT |
|  | R: GAGTCCCGGAATGTTGCAGT |
| q-FABP4 | F: TGAGATTTCCTTCAAATTGGG |
|  | R: CTTGTACCAGAGCACCTTCATC |
| q-ELOVL6 | F: AACACCGATGTGGATTGCCT |
|  | R: CAGCTCATCCTCCGACTTGG |
| m6A-PPARGC1B | F: GAAGCTGTGATGTTGGTGCC |
|  | R: CAGTGTCATACTCGGGGCTC |
| m6A-ADIPOQ | F: CACTGTCCCCAATGTTCCCA |
|  | R: GCCAGTGGTGCCATCATAGT |
