# Supplementary material for: Dynamic m⁶A methylation during bovine preadipocyte differentiation and functional implication of the m⁶A writer METTL14
Source: BMC Genomics. 2026 Apr 20;27:511. doi: 10.1186/s12864-026-12858-w (PMC13224485; doi:10.1186/s12864-026-12858-w)
Supplement: Supplementary file 2 — Supplementary Material 2. [file 12864_2026_12858_MOESM2_ESM.docx]

**The intersection of key m⁶A-modified genes in the early stage of differentiation and genes significantly altered by METTL14 knockdown.**

SPP1, TNC, ASPH, CSTB, DKK2, H1-2, ARHGDIB, WNT4, NKD1, SPRY4, PPARGC1B, PMEPA1, ST8SIA4, MMRN2, BANK1, ZBTB46, ARHGAP26, ATXN7L1, B4GALNT1
