# Supplementary material for: Dynamic m⁶A methylation during bovine preadipocyte differentiation and functional implication of the m⁶A writer METTL14
Source: BMC Genomics. 2026 Apr 20;27:511. doi: 10.1186/s12864-026-12858-w (PMC13224485; doi:10.1186/s12864-026-12858-w)
Supplement: Supplementary file 3 — Supplementary Material 3. [file 12864_2026_12858_MOESM3_ESM.docx]

**The intersection of key m⁶A-modified genes during the lipid deposition stage and genes significantly altered by METTL14 knockdown.**

TF, SPP1, NDRG2, POSTN, TNC, TGFBI, UVRAG, PLPP3, NT5E, ADIPOQ, MT2A, LPIN1, VCAM1, MT1A, MCAM, LIPE, ADCY5, ATP2C2, CREB5, VAT1L, NGFR, PLIN4, DKK2, PVR, SUCNR1, AOC3, WNT2B, OGN, SAT2, WNT4, SPRY4, TNFSF13B, PMEPA1, ST8SIA4, DHX58, BANK1, ARHGAP26, BHLHE41, RNASE10, SPON2, TMEM51, PLCB1, PARD6A
